# Characterization of four subtypes in morphologically normal tissue excised proximal and distal to breast cancer

**DOI:** 10.1038/s41523-020-00182-9

**Published:** 2020-08-21

**Authors:** Emanuela Gadaleta, Pauline Fourgoux, Stefano Pirró, Graeme J. Thorn, Rachel Nelan, Alastair Ironside, Vinothini Rajeeve, Pedro R. Cutillas, Anna E. Lobley, Jun Wang, Esteban Gea, Helen Ross-Adams, Conrad Bessant, Nicholas R. Lemoine, Louise J. Jones, Claude Chelala

**Affiliations:** 1 Centre for Cancer Biomarkers and Biotherapeutics, Barts Cancer Institute, Queen Mary University of London, London, EC1M 6BQ UK; 2 Centre for Computational Biology, Life Sciences Initiative, Queen Mary University of London, London, EC1M 6BQ UK; 3 Centre for Tumour Biology, Barts Cancer Institute, Queen Mary University of London, London, EC1M 6BQ UK; 4 Center for Cancer Genomics and Computational Biology, Barts Cancer Institute, Queen Mary University of London, London, EC1M 6BQ UK; 5 School of Biological and Chemical Sciences, Queen Mary University of London, London, E1 4NS UK; 6 Barts Cancer Institute, Queen Mary University of London, London, EC1M 6BQ UK

**Keywords:** Breast cancer, Tumour biomarkers

## Abstract

Widespread mammographic screening programs and improved self-monitoring allow for breast cancer to be detected earlier than ever before. Breast-conserving surgery is a successful treatment for select women. However, up to 40% of women develop local recurrence after surgery despite apparently tumor-free margins. This suggests that morphologically normal breast may harbor early alterations that contribute to increased risk of cancer recurrence. We conducted a comprehensive transcriptomic and proteomic analysis to characterize 57 fresh-frozen tissues from breast cancers and matched histologically normal tissues resected proximal to (<2 cm) and distant from (5–10 cm) the primary tumor, using tissues from cosmetic reduction mammoplasties as baseline. Four distinct transcriptomic subtypes are identified within matched normal tissues: metabolic; immune; matrisome/epithelial–mesenchymal transition, and non-coding enriched. Key components of the subtypes are supported by proteomic and tissue composition analyses. We find that the metabolic subtype is associated with poor prognosis (*p* < 0.001, HR6.1). Examination of genes representing the metabolic signature identifies several genes able to prognosticate outcome from histologically normal tissues. A subset of these have been reported for their predictive ability in cancer but, to the best of our knowledge, these have not been reported altered in matched normal tissues. This study takes an important first step toward characterizing matched normal tissues resected at pre-defined margins from the primary tumor. Unlocking the predictive potential of unexcised tissue could prove key to driving the realization of personalized medicine for breast cancer patients, allowing for more biologically-driven analyses of tissue margins than morphology alone.

## Introduction

Improved mammographic screening and increased self-monitoring allows for the detection of breast cancer in asymptomatic stages. This has led to an increased trend toward breast-conserving treatment (BCT) often with adjuvant radiotherapy^1,2^.

Completeness of excision is currently based on histological examination of surgical margins. Despite apparently clear margins, ipsilateral recurrence is reported in over 40% of patients receiving BCT alone and in up to 20% of patients receiving adjuvant radiotherapy^2,3^. It is not clear whether recurrence is attributable to missed residual microscopic disease or to the development of further perturbations within the unexcised field of cancerization.

Perceived risk status, determined primarily by pathological evaluations and genomic tests focusing on the tumor, is used to guide the therapeutic management of patients. However, novel lines of evidence indicate that histologically normal (HN) tissue resected adjacent to primary tumors has prognostic capabilities, with about 40% of these tissues exhibiting aberrant genomic features and behavior^4–6^.

These conclusions are usually based on pan-cancer studies that, while providing a comprehensive overview of similarities and differences across cancer types, may lack the granularity offered by cancer-specific research. Data from The Cancer Genome Atlas (TCGA)^7^, often used as a training or validation cohort, provides access to invaluable data generated from different platforms and sample types. However, detailed information about the spatial location of cancer-adjacent tissues is not provided. Cancer-adjacent specimens are described by the TCGA as tissue resected ≥2 cm from the tumor margin and defined as morphological normal by histopathologic assessments. As such, researchers are not able to analyze any distance-related effects using this dataset. Finally, key studies on cancer-adjacent tissues do not investigate the therapeutic implications of their findings^4–6^.

Our study provides insight into molecular alterations in HN tissues within the affected breast and offers a greater understanding of the spatial implications of these aberrations. This was achieved by performing a comprehensive analysis of RNA sequencing data and proteomic profiles from breast cancers (*n* = 19) and their matched HN tissues (*n* = 38), healthy breast from cosmetic reduction mammoplasty (RM; *n* = 5), and risk reducing mastectomies (RR, *n* = 5), with peritumoral samples excised at proximal (TP, <2 cm) and distal (TD, 5–10 cm) sites from the primary tumor.

We compared the transcriptomic profiles of matched HN tissues to those of healthy breast and identified alterations in matched tissues resected up to 10 cm from the primary tumor. Applying unsupervised classification to the transcriptomic expression profiles of all HN tissues resolved four distinct transcriptomic subtypes that are independent of distance from the primary tumor: termed metabolic, immune, matrisome/epithelial–mesenchymal transition (matrisome/EMT) and non-coding (nc) enriched. We then integrated our transcriptomic findings with those from proteomic/phosphoproteomic analyses (*n* = 33) to explore the prognostic and therapeutic potential of our subtypes.

The ability to prognosticate outcome or predict response to therapy from morphologically normal tissues in women with breast cancer could help improve prognostic and therapeutic determinations in breast cancer. Our findings raise the proposition that breast cancers should be evaluated in conjunction with their surrounding tissue, with completeness of excision being determined by both molecular and morphological features.

## Results

### Cohort characteristics

**Fig. 1:**
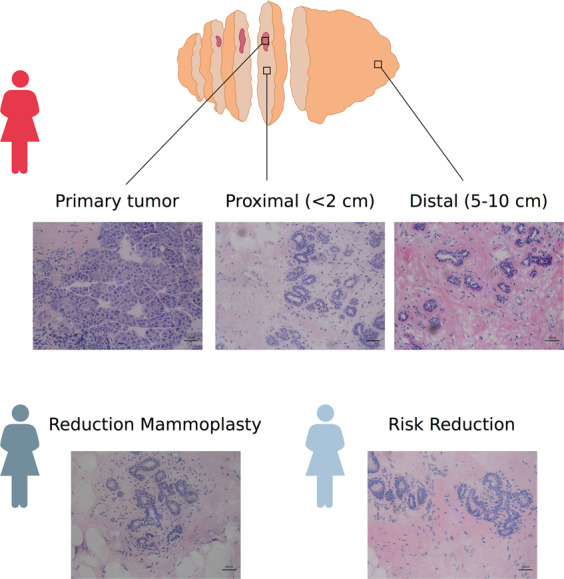
Representative immunohistochemical images and locations of sample types. Immunohistochemical images representing tissues collected from tumor, TP, TD, RR, and RM. Images shown at ×20 magnification, with scale bars at 50 μm.

Fresh tissue samples were collected prospectively from breast cancer patients (*n* = 57) and age-matched risk reduction (*n* = 5) and reduction mammoplasty (*n* = 5) patients, following informed patient consent. Fresh breast tissue specimens were assessed macroscopically and, in cases of breast cancer, tumor and non-tumor areas were identified. Samples from tumor and macroscopically normal tissue proximal to (TP, <2 cm) and distant from (TD, 5–10 cm) the tumor were collected as individual frozen tissue blocks (Fig. 1). Histopathological characterization of both tumor and non-tumor tissue was then performed for each block. In addition, key pathological characteristics of the invasive tumor and any relevant benign breast disease present in the non-tumor tissue, including those sampled from the risk reducing and cosmetic reduction specimens, were recorded (Supplementary Data Set 1). Data associated with one RM patient did not pass initial quality assessments and so this patient was removed from further analyses.

### Matched HN tissues adjacent to cancer exhibit heterogeneity in their transcriptomic profiles

Principal component analysis (PCA) of the top 5000 most variable genes shows the first principal component to reflect malignancy (Supplementary Fig. 1). The tumor profiles and RM profiles aggregate in distinct areas of transcriptional space, with the latter displaying greatest homogeneity in transcriptomic profiles relative to all other groups. In contrast, the expression profiles of morphologically normal samples (TP and TD) display great heterogeneity.

### Matched HN tissues adjacent to cancer display tumor-associated characteristics

The PAM50 single-sample predictor^8^ was applied to both the tumor and normal data to offer an indication into potential tumor characteristics exhibited by the specimens (Supplementary Data Set 2 and Supplementary Fig. 2).

RM samples are defined as normal-like, however, tumor-like features are observed in HN tissues. While the number of samples assigned to the normal-like subtype in HN profiles derived from luminal tumors increases with distance from primary tumor (tumor = 0, TP = 4, TD = 8), the inverse is true for the matched profiles from triple negative tumors, with the majority of normal-like tissues reported in the TP group (tumor = 0, TP = 3, TD = 1).

In agreement with previous literature, the transcriptomic profiles of luminal tumors (9 out of 13), as determined by IHC and molecular subtyping, are categorized as either luminal or normal-like by PAM50^6^. However, the intrinsic subtype allocation of HN tissues is not always dependent on the associated primary tumor.

We evaluated whether matched normal tissues were able to predict overall survival based on PAM50 intrinsic subtype. TCGA cancer-adjacent samples were stratified based on their PAM50 molecular subtype (Luminal A, *n* = 33, Luminal B, *n* = 11, Basal-like, *n* = 31, Her2-enriched, *n* = 13, and Normal-like, *n* = 20) and univariate Cox regression analysis performed. We found that the molecular subtype in this cohort was not significantly associated with outcome (log-rank *p* > 0.1).

### Cancer-associated aberrations are present in proximal and distal tissues

Differential expression (DE) analysis was conducted to identify aberrant features in tumors and matched tissues. By using RM as a baseline, we adjusted for confounding transcriptomic influences present in matched breast tissues.

The total number of DE genes decreases in relation to distance from primary tumor (FDR ≤ 0.05 and a log-fold change of ≥1) (Supplementary Fig. 3), with the trend of upregulated genes being greater than downregulated genes across each comparative group (tumor: upregulated *n* = 1841, downregulated *n* = 1312; TP: upregulated *n* = 247, downregulated *n* = 121; TD: upregulated *n* = 264, downregulated *n* = 102).

To elucidate fundamental pathways disrupted in these tissues, we used the pathway databases KEGG and Reactome, defined GO terms and performed GSEA analysis, using the 50-hallmark gene sets (GSEA false discovery rate [FDR] *q*-value < 0.05)^9^.

Pathways previously linked to cancer are highlighted in the examination of tumor samples (Supplementary Fig. 4). The most highly enriched functional categories include those associated with proliferation and cell cycle progression, cancer-associated signaling pathways and immune response.

In agreement with previous publications, the main pathways affected in TP tissues include cellular response to external stimuli and chemotaxis^4–6^. However, the most prominent observation is the consistent dysregulation of metabolic pathways, such as those involved in lipid metabolism and xenobiotic metabolic processes. The most significant results from GSEA analysis are EMT, fatty acid metabolism, androgen response, cancer-associated signaling pathways mTORC1, and coagulation. Cellular response to external stimuli (heat stress response), extracellular matrix organization, and movement continues to be affected in TD tissues.

Overall, our DE analysis reports that morphologically normal tissues resected proximal to and distant from cancer exhibit molecular alterations compared to the RM tissues.

### Four transcriptional subtypes are identified in matched HN tissues

Unsupervised non-negative matrix factorization (NMF)^10^ of the RNA-seq data identified two classes (cophenetic coefficient 1) representing the tumor and “normal” phenotypes (Supplementary Fig. 5a). Profiles of matched normal samples (TP, TD, RR, and RM) were subsequently assessed independently of tumor profiles to gain a greater understanding of the heterogeneity of these tissues.

**Fig. 2:**
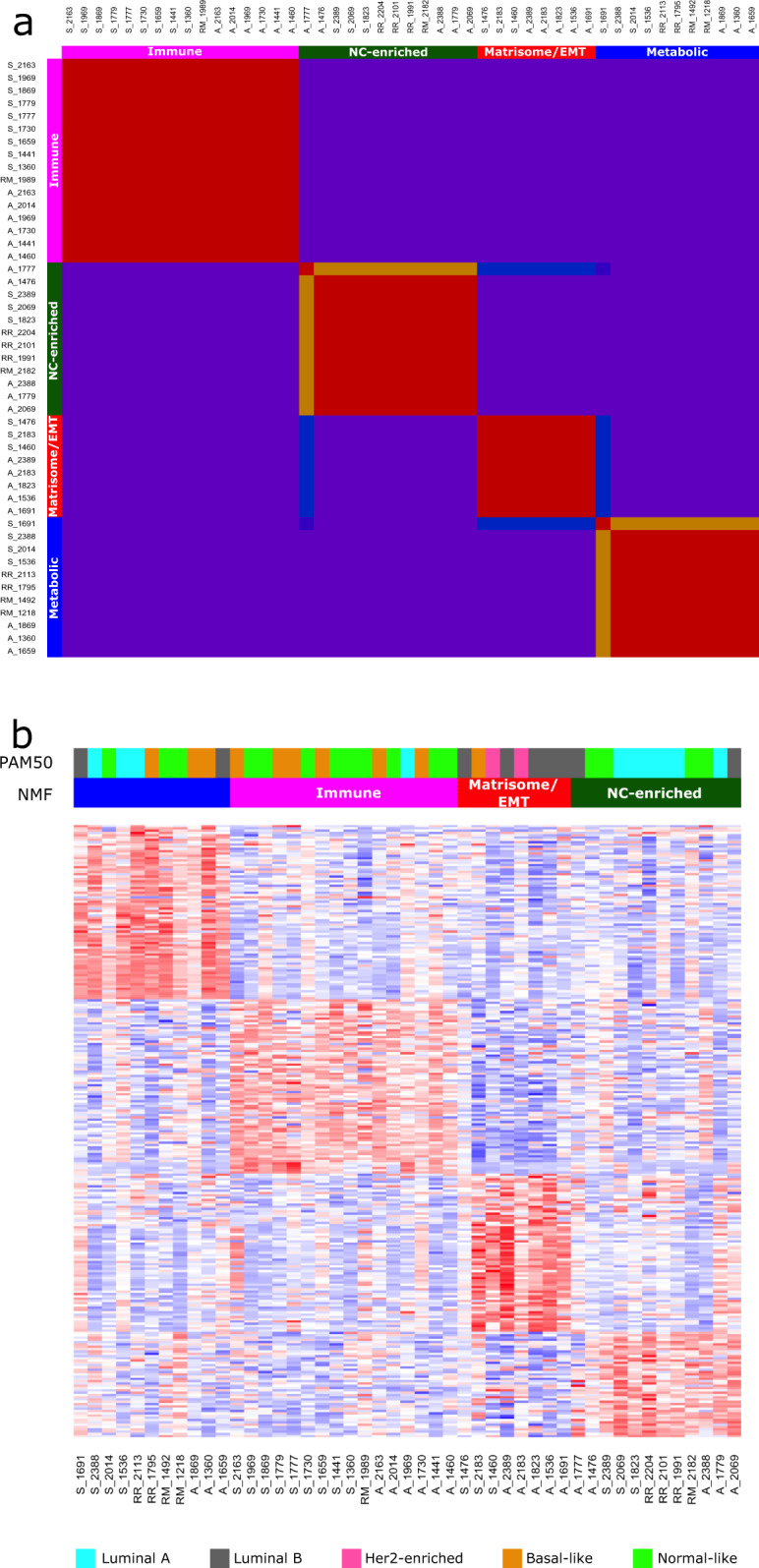
Four subtypes are identified in matched HN tissues. **a** NMF on data derived from morphologically normal tissues resolves into four clusters (cophenetic coefficient 0.9991). **b** Heatmap of normalized abundance for genes defined as highly differentially abundant between the classes in a SAMseq multiclass analysis reveals the distinct transcriptomic patterns between the subtypes.

NMF classification of HN transcriptomic profiles identifies four classes in the samples (cophenetic coefficient 0.9991) (Fig. 2a and Supplementary Fig. 5b) that remain stable even with the exclusion of RM and RR samples (cophenetic coefficient 0.9955). These four clusters are defined using an aggregate of all tissues and, as such, are independent of distance from the primary tumor.

We generated an abundance heatmap for the selected clustering solution by identifying a 414-gene classifier: genes that were differentially abundant between the clusters (Fig. 2b and Supplementary Data Set 3). These subtypes were defined and named according to their classifier attributes and pathway enrichments: (i) metabolic; (ii) immune; (iii) matrisome/EMT; and (iv) nc-enriched.

To validate these subtypes and identify potential therapeutic targets, phosphoproteomic profiling was performed on a subset of tumors (*n* = 11) and their matched specimens (*n* = 22). This was followed by kinase substrate enrichment analysis (KSEA), which infers kinase activity by matching phosphorylation sites to known upstream kinases^11^.

### Metabolic subtype

**Fig. 3:**
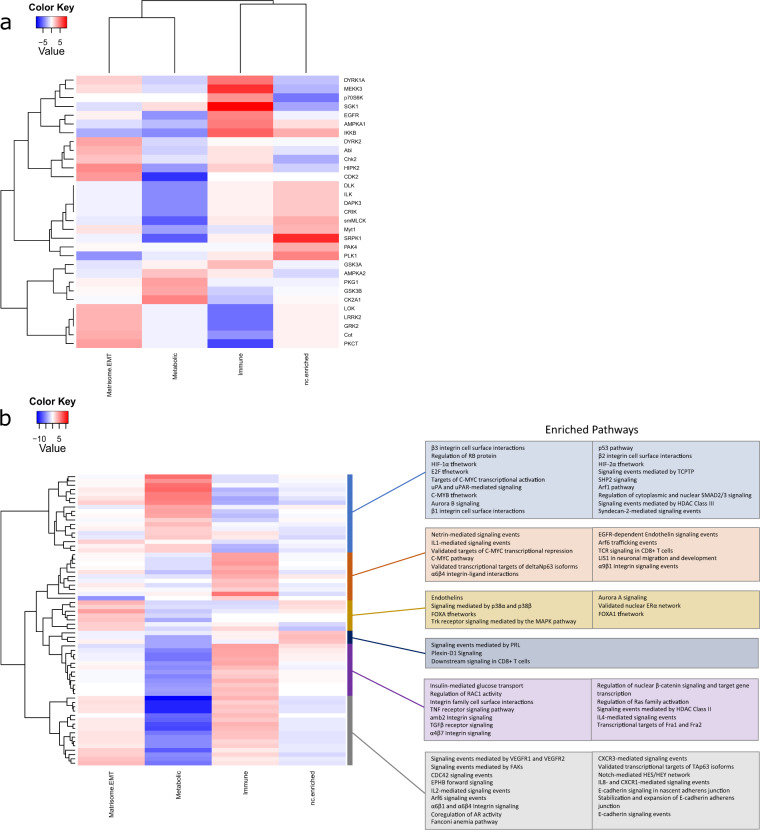
The phosphoproteomic profiles of tissues in each transcriptomic group exhibit altered phosphorylation and kinase activities in proteins associated with distinct biochemical and signaling processes. **a** Diverse substrate groups differentially enriched between the subtypes. **b** Heatmap of the top kinase pathways enriched in the subtypes. A complete list of pathways is available from Supplementary Data Set 4.

The metabolic subtype (*n* = 11) exhibits the greatest global deregulation of its transcriptomic and phosphoproteomic signature relative to the immune, matrisome/EMT and nc-enriched subtypes (Fig. 3 and Supplementary Data Set 4). This deregulation is focused on mediators of metabolic processes, lipid and cholesterol metabolism, and hypoxia-related events. This is exemplified in the enrichment of cancer-associated metabolic and proliferative events, such as integrin signaling, IF1/2-mediated events, Syndecan-2-mediated signaling and uPA/uPAR-mediated signaling, and MYC and associated transcription factors. Furthermore, the premise that our metabolic subtype is associated with cellular metabolism is reinforced by overrepresentation of genes and pathways that regulate members of the solute carrier (SLC) and ATP-binding case, in particular those induced by hypoxia and those that support the movement of glucose. Phosphorylation analysis identifies kinase activations sites in potential kinase drug targets, such as AURKB, CDK5, and GSK-3β^12–15^.

Our findings indicate that aberrations to the metabolic circuitry are not localized to the primary tumor or its immediate vicinity but that they can extend to distal tissues. This presents a potential therapeutic target for patients whose tissues are allocated to this subtype.

### Immune subtype

The main genes, terms and pathways enriched in the HN tissues of the immune subtype (*n* = 16) suggest activation of the immune response. Phosphoproteomic profiling and kinome substrate enrichment also indicate overrepresentation of immune-related characteristics (Fig. 3 and Supplementary Data Set 4). The insulin pathway and α6 integrins are involved with immune surveillance and immunometabolic response^16,17^. Similarly, activation of kinase substrates associated with inflammatory and immune responses are present (SGK1, IKKB, p70S6K, HIPK2).

### Matrisome/EMT subtype

This subtype (*n* = 8) is enriched in matrisomal and EMT elements, which collaborate with matrisome-affiliated proteins, regulators and secreted factors to regulate cell behavior and provide cues fundamental to proliferation, differentiation, and migration. In conjunction with a range of genes linked to functionalities of the matrisome, our analysis reports elevated levels of mRNA for *KIF14*, *KIF18B*, *TUBA3D*, and *TUBA3E* and other components of the microtubule organization support mechanism. These transcriptomic observations are supported by the proteomic analysis in which an enrichment of activities associated with angiogenesis and the metastatic cascade are reported, these include E-cadherin, endothelin, pathways involved in TGF-β1 signaling (SMAD dependent and SMAD independent) and Aurora A signaling (Fig. 3 and Supplementary Data Set 4). Potential kinase-targeted therapy targets identified in this group include MAP3K8, GRK2, and AURKA. Interestingly, osteopontin-mediated events and associated pathways, previously linked to cancer progression, are depleted in both the matrisome and the metabolic subtypes^18,19^.

### Non-coding enriched subtype

The nc-enriched subtype (*n* = 12) comprises 61.3% (57/93) nc elements. This subtype is enriched for antisense (*n* = 10), lincRNA (*n* = 15), pseudogenes (*n* = 18), and sense intronic (*n* = 7) genes. The functions of these genes have yet to be determined, however, the regulatory targets of these elements may yield further insight into the phenotypic character of this class. Literature mining of the ncRNAs found 29 of these elements to have been reported previously in cancer studies, but not in matched HN tissues, while 28 of these elements are novel (Supplementary Data Set 5). The proteomic profile of the nc-enriched subtype exhibits low-level alterations.

### Functional characteristics of the translated elements associated with HN tissues recapitulate the transcriptomic subtypes

Proteomics informed by transcriptomics (PIT) is a method by which spectra from liquid chromatography tandem mass spectrometry (LC-MS/MS) and the de novo transcriptome assembly are used to infer all translated genomic elements (TGEs) in a sample^20^.

This methodology is sample-specific and is not dependent on a standard database for mass spectrometry peptide identification. This allows for the identification of novel translated elements, such as polymorphisms, alternative splicing events, products of genomic regions previously thought to be non-coding, as well as unknown proteins.

**Fig. 4:**
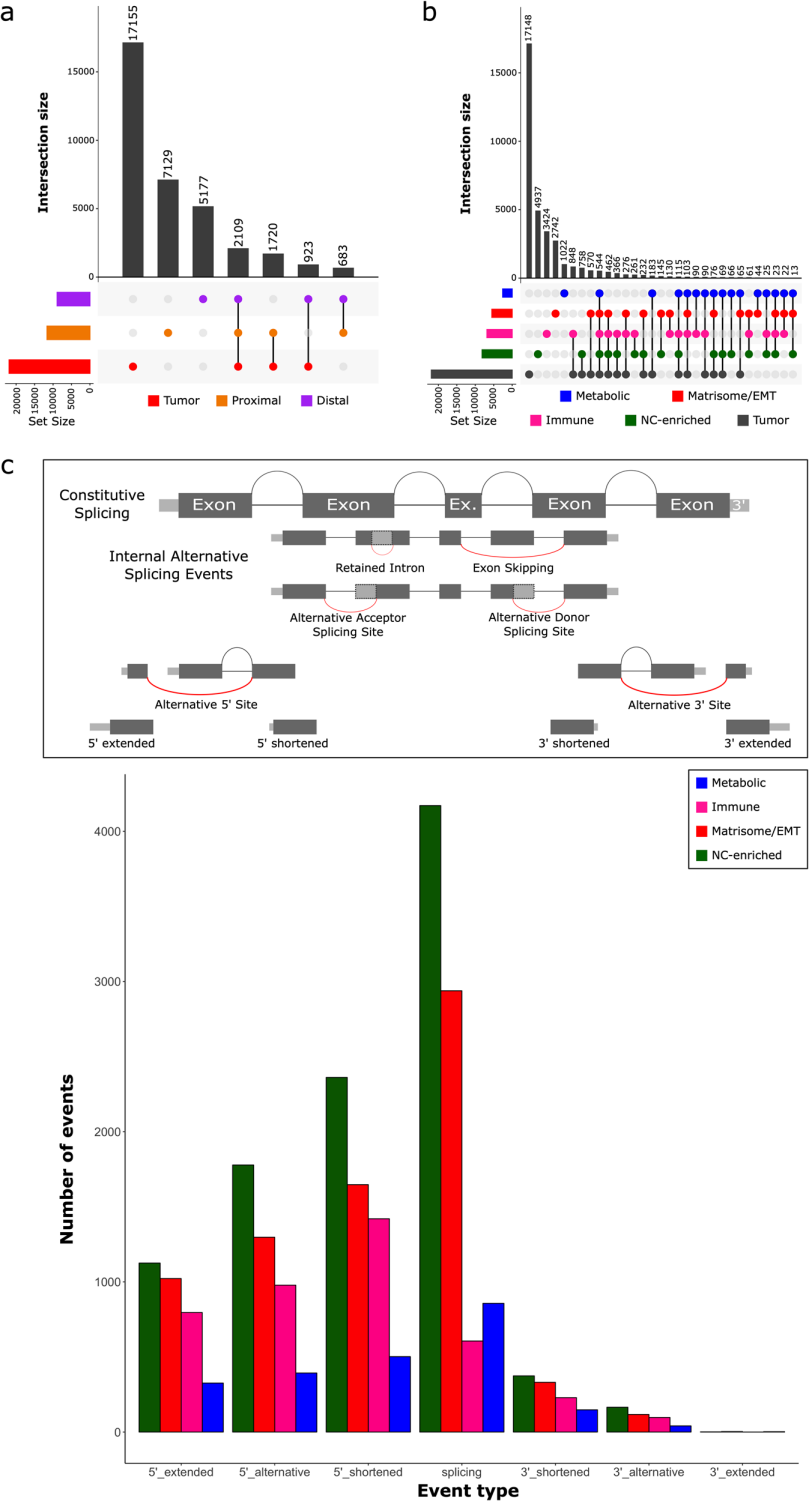
PIT detects translational patterns in the data. The rows in the UpSet represent the spatial location or subtype being assessed and the columns represent the intersections and aggregates of conditions. The bar plot displays the number of translated elements identified for each intersection or aggregate. **a** The number of TGEs decreases with distance from the primary tumor, with translated elements being reported common between both HN subtypes and tumor. **b** The translational profiles of the transcriptomics-derived subtypes differ. While they exhibit distinct characteristics, all of the HN profiles also show commonalities with each other and with the tumor profile. **c** The nc-enriched subtype is enriched for TGE linked to alternative splicing events.

PIT analysis of the orientation of the specimens reports that the total number of TGEs decreases with distance from the primary tumor (Fig. 4a). As observed in the transcriptomic analysis, overlap between tumor features is observed within TP and TD tissues.

Each transcriptomic subtype exhibits both distinct and common translated features (Fig. 4b). Closer inspection of the functionalities and GO terms reported as uniquely enriched in each subtype are in agreement with the transcriptional interpretation of the HN subtypes.

Unlike standard proteomic profiling, PIT allows for greater characterization of data pertaining to the nc-enriched subtype. The defining features of this subtype are associated with alternative splicing categories within the PIT dictionary (Fig. 4c), with an enrichment of pathways and GO terms associated with assembly of the spliceosomal complex, oxidative phosphorylation, and regulation of mRNA processing.

**Fig. 5:**
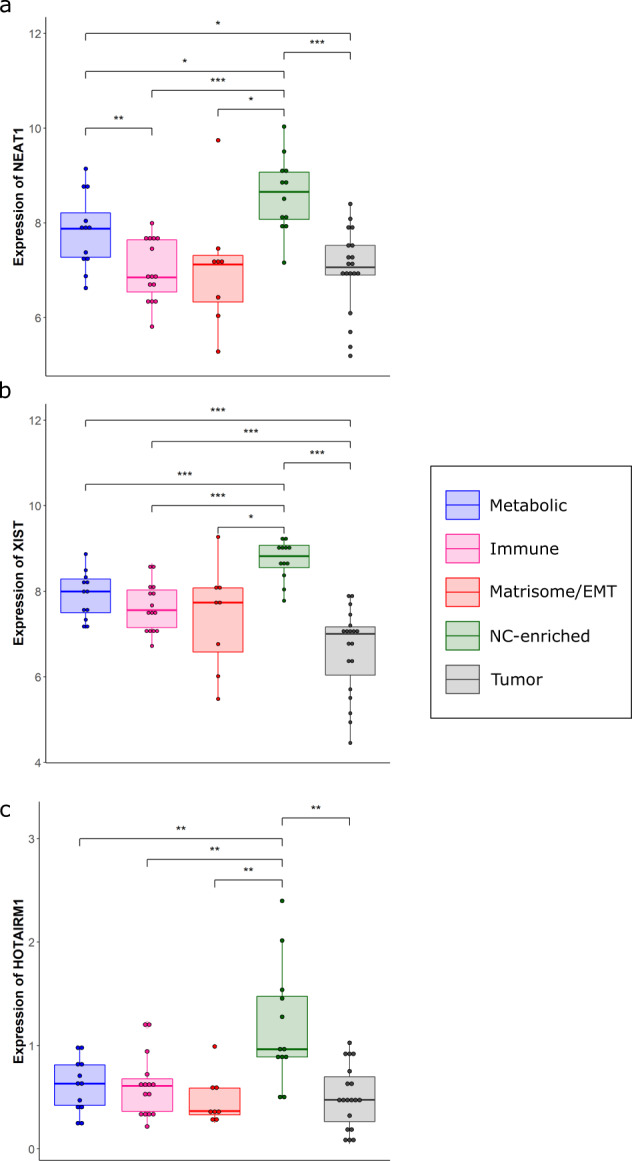
Boxplots showing the non-negative expression values of three well-known ncRNAs associated with alternative splicing across all HN subtypes and primary breast cancer. The central line in the boxes represent the median expression value, the boundaries of the boxes represent the interquartile range and the ends of the whiskers represent the minimum and maximum values in the data. The expression of NEAT1 **a**, XIST **b**, and HOTAIRM1 **c** are significantly higher in the nc-enriched subtype relative to the remaining HN subtypes and in primary tumors.

This validates our transcriptomic findings in which a number of ncRNAs that regulate alternative splicing are overexpressed in the nc-enriched subtype. These include NEAT1, HOTAIRM1, and XIST, which interact with splicing factors and/or influence chromatin remodeling (Fig. 5).

### Immune composition of the transcriptional subtypes suggests the presence of tumor‐associated macrophages in the metabolic subtype

**Fig. 6:**
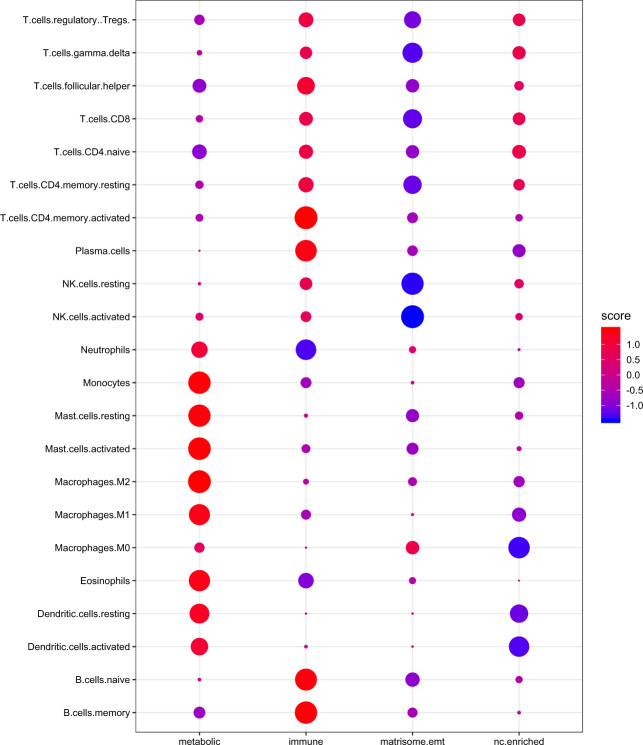
Deconvolution analysis of the transcriptional subtypes. Each subtype has unique features of cellular composition.

Diversity within the HN tissues led us to hypothesize that each subtype may exhibit characteristics related to cellular composition. We implemented CIBERSORT^21^, using the validated LM22 signature matrix, to reveal patterns in the immune characteristics of each subtype (Fig. 6). This matrix consists of 547 genes capable of differentiating 22 human hematopoietic cell populations. These include seven types of T cells, naive and memory B cells, plasma cells, NK cells, and myeloid subsets. Inter-group normalization was also performed to allow for the immune profile of each subtype to be evaluated in relation to each other.

The metabolic subtype is enriched for myeloid populations while the immune subtype is enriched for lymphoid populations, in particular components recruited in inflammatory response and adaptive immunity, such as CD4+ T cells and B cells. The nc-enriched subgroup exhibits a pattern of immune composition that tends to inversely mirror that of the metabolic group. Finally, the matrisome/EMT group does not appear to have defining immune characteristics. It is depleted in almost all categories pertaining to the lymphoid group relative to the other subtypes.

Our transcriptomic and proteomic findings identify multiple entities associated with increased TAM density in the metabolic subtype. These include high expression of *ADIPOQ*, *AGPAT2*, *IL6*, *NDUFA4L2*, and *S1PR1* at the transcriptomic level (Supplementary Data Set 3) and dysregulation of the HIF1/2-α pathways, integrin cell surface markers (β1 and β3) and osteopontin-mediated events at the proteomic level (Fig. 3)^22–24^.

We provide an overview of immune composition in each subtype. It is important to consider that CIBERSORT infers immune features using bulk tissue and that this may limit the potential implication of the findings. In addition, information about the distribution patterns of immune cells is not provided. The localization of macrophages and tumor-infiltrating lymphocytes is key to determining their effects. As such, these findings should be interpreted with care.

### The metabolic subtype exhibits poor prognosis

**Fig. 7:**
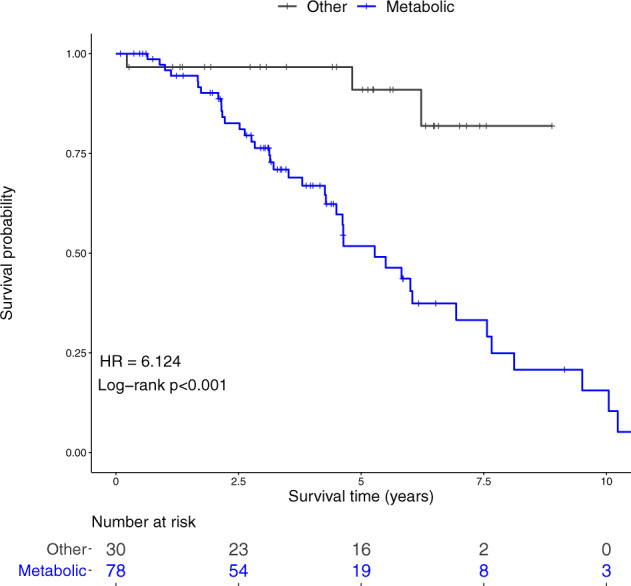
Kaplan–Meier plot showing the relationship between the metabolic subtype and outcome. Cancer-adjacent samples from the TCGA BRCA cohort was used to estimate the prognostic value of our findings. Each cancer-adjacent sample from the TCGA was assigned to a transcriptomic subtype. Patients allocated to the metabolic risk group have a worse prognosis relative to patients in all other risk groups (log-rank *p* < 0.001, hazard ratio 5.8). The hazard ratio was estimated using a Cox proportional hazards model, and curves were compared using a log-rank test.

In the absence of publicly available datasets with similar sampling design and sufficient specimens, we selected 108 matched adjacent tissues from the TCGA BRCA dataset to validate our subtypes. Support vector machine (svm)-based modeling was applied to assign each TCGA adjacent sample to one of our transcriptomic subtypes. We were able to classify the TCGA samples into three of our four subtypes— metabolic, immune, and nc-enriched. The matrisome/EMT subtype, as defined by our study, was not found within the TCGA cohort. Univariate Cox regression analysis was applied to determine the prognostic value of the metabolic signature (*n* = 78). We report patients whose HN tissues are assigned to the metabolic subtype to exhibit poor prognosis (log-rank *p* < 0.001, HR6.1) (Fig. 7 and Supplementary Fig. 6).

While the sample size of the immune and nc-enriched risk groups is too small to make meaningful conclusions (*n* ≤ 16), the trends appear to suggest that these are associated with good prognosis, with no events recorded in the nc-enriched subtype. However, further investigations are needed to validate the prognostic implications of these observations.

Univariate modeling of our 414-gene signature identified 34 genes with significant prognostic potential (log-rank *p* < 0.01) in HN tissues (Supplementary Data Set 6). Among the most prognostic genes are *SLC2A4*, *LGALS12*, *MRC1*, and *G0S2*, all of which are genes that define the metabolic signature.

Secondary univariate modeling stratifying the TCGA dataset into two groups: age-matched (≤55) and older (>55 years) support the premise that these genes have greater predictive value in the younger cohort (Supplementary Data Set 6). However, the low sample size available for validation in the younger group makes confident determinations unfeasible.

## Discussion

This study applies an integrated multidisciplinary approach to take an important first step toward characterizing matched normal tissues resected at pre-defined margins from the primary tumors. Our findings support the proposition that histological normalcy does not imply biological normalcy. Defects at margins of resection of 4 cm have previously been reported^4,6^. However, we observe alterations in tissues located up to 10 cm from the primary tumor.

We identify four distinct transcriptomic subtypes in HN tissues of young women with breast cancer; termed metabolic, immune, matrisome/EMT, and nc-enriched. We also find that it is the molecular characteristics of the HN tissues within each subtype, rather than their distance from primary tumor, that has the greatest predictive value.

All specimens allocated to the metabolic subtype exhibit disruptions to their metabolic circuitry. Metabolic reprogramming, mediated by cross-talk between signaling pathways, hypoxia, and metabolic networks, has been shown to remodel the microenvironment and drive proliferation, tumorigenesis, progression, and resistance to treatment^25,26^. Our findings indicate that the disruptions to cellular genetics observed in this subtype result in poor prognosis. The most prognostic genes identified in our gene signature map to the metabolic subtype and have been recognized as prognostic or therapeutic factors in cancer but not matched normal tissues^27–30^. To the best of our knowledge, this is the first study to identify these prognostic genes in matched normal tissues.

Deconvolution analysis noted a heavy enrichment of myeloid cells and M1/M2 macrophages in this subtype. The enrichment of myeloid cells in cancer-adjacent tissues has been reported previously^4^. However, unlike studies that examine cancer-adjacent tissues as a whole, we observe this phenomenon primarily in the metabolic subtype. Recent studies also indicate that M1 and M2 macrophages promote an immunosuppressive phenotype when exposed to distinct microenvironmental cues, such as hypoxia^22^. This macrophage profile could suggest the presence of tumor‐associated macrophages (TAMs), which are associated with poor prognosis and generating a hypoxic pre-metastatic niche^31,32^.

Promising actionable metabolic therapeutic targets, such as AURKB, CDK5, and GSK-3β, are enriched in this subtype. This, in conjunction with findings from previous research, suggests that the metabolic rewiring process could be susceptible to metabolic therapy^12–15^.

HN tissues allocated to the immune subtype display activation of an immune environment, which is consistent with recent findings^4^. This appears to play a protective role, with patients allocated to this group having good prognosis. High rates of lymphocyte infiltration in colon, ovarian, and ER-negative breast cancer have been associated with increased survival and better pathological response rates relative to their low-density counterparts^33,34^. In addition, recent investigations report that the spatial architecture of tumor-infiltrating lymphocytes is highly predictive of recurrence^35^. As such, it would be informative to study the behavior and localization characteristics of the immune cells present in tumors and matched HN tissues of this subtype to help refine individual prognostication.

All samples in the matrisome/EMT subtype exhibit imbalances between cell–matrix and cell–cell adhesion pathways and disruptions to matrisome processes that provide biophysical and biochemical cues fundamental in regulating of EMT pathways and rigidity of the extracellular matrix (ECM). ECM rigidity is proving vital in regulating tumor spread and metastasis, and in the context of mammographic density, increases the risk of breast cancer. Furthermore, ECM rigidity and fibrotic co-localization has been recognized as a prognostic marker of distant metastasis in breast cancer, and chemoresistance to paclitaxel in pancreatic cancer^36,37^.

We report similarities between genes defining our matrisome/EMT subtype and matrisome gene sets reported previously in breast, prostate, bladder, and ovarian cancer studies^38,39^. Unlike these studies, we find that disruptions to the matrisome are already present in matched HN tissues, suggesting that these tissues could, themselves, have prognostic and therapeutic potential. For instance, MAP3K8 and AURKA identified by our phosphoproteomic interpretations are predictive biomarkers for treatment efficacy and mediators of anti-tumor activities in solid cancers^40,41^. Interestingly, we also observe dysregulation of osteopontin-mediated events, which is a key player in creating an immunosuppressive and pro-tumorigenic microenvironment, and is reported as a prognostic marker in a range of solid tumors^18,19^.

About 93% of the human genome is actively transcribed. However, <2% of the human genome encodes proteins, with at least 75% being transcribed into ncRNA. Understanding the functions of nc regulatory elements has gained considerable traction recently but their mechanisms of action remain elusive. All patients in the TCGA validation cohort allocated to the nc-enriched subtype (*n* = 16) did not suffer cancer-related deaths over 10 years, suggesting that this group is defined by a non-malignant signature.

Transcriptional elements identified in this subtype have been previously reported for their tumor suppressor capabilities^42–44^. In addition, nc elements have been found to regulate alternative splicing, which supports our transcriptomic and PIT observations. Key TGEs defining this subtype, such as NEAT1, interact with splicing factors and influence chromatin remodeling. Unlike pan-cancer studies that report low expression of NEAT1 in breast tumors relative to their corresponding HN matched tissues, we observe this phenomenon only in samples pertaining to the nc-enriched and, to a lesser extent, the metabolic subtype^45^. This suggests that NEAT1 is overexpressed in HN tissues from a subset of patients rather than all HN tissues ubiquitously as reported previously.

The metabolic, immune and nc-enriched subtypes are recapitulated in the TCGA cohort, with this external dataset identifying the metabolic subtype as having poor overall survival. The matrisome/EMT subtype is notably absent in this cohort possibly due to differences in sample collection and patient characteristics.

TCGA samples do not accurately reflect the breadth of HN samples used in our study. Cancer-adjacent samples available from the TCGA cohort are resected at margins >2 cm from the primary tumor, meaning that the TCGA collection is not able to represent our TP group. With five out of nine samples in the matrisome/EMT subtype being TP, this may explain, in part, why this subtype was not reproduced in the validation cohort.

Patient determinants, such as age and menopausal status, have been reported independent discriminants for breast cancer recurrence^46^. Our patient cohort comprises specimens from 19 young breast cancer patients (mean 41.3 years; range 32–53 years) and controls from RR and RM patients (mean 36 years; range 22–48). The TCGA cohort comprises tumor-normal matched specimens obtained from a wide range of ages (mean 57 years; range 30–90 years). Matching the age profile between TCGA and our data reduces the number of TCGA samples to 50 patients (≤55 years), offering insufficient samples to power subsequent analyses.

Young age has been shown to be an independent negative prognostic factor for breast cancer recurrence and survival. The difficulty of obtaining age-specific cohorts is likely why prognostic models in young women often produce suboptimal results. Our research highlights this paucity of data available from young breast cancer patients and suggests that further research is warranted to determine the prognostic and therapeutic significance of any differences.

Limitations of this study include low power due to the small cohort available with specimens available from primary tumor, TP, and TD tissues. However, concordance of key transcriptomic findings from deconvolution and proteomic analyses increase the confidence of our results despite the small sample size. Ideally, validation is performed using independent studies that have been sampled in the same manner. Our sampling design was not mirrored in publicly available datasets and so direct reproduction of the workflow was not possible. Instead, the TCGA BRCA cohort was used for validation, but, as discussed previously, the differences in sampling design and patient covariates may have prevented complete recapitulation of our findings. While our work is indicative of key transcriptomic and proteomic changes in TP and TD tissues, further investigations are warranted to reveal the full significance of these alterations.

Our findings support the premise that breast cancer biology should encompass the mechanistic roles of all cell types within the affected breast. The identification of distinct molecular subtypes in HN tissue from women with breast cancer has a number of clinically relevant implications: signatures that predict poor prognosis, such as the metabolic subtype, could be used to tailor enhanced surveillance for patients undergoing BCT. Elucidating the dynamics underlying the cross-talk between tumor and matched normal tissue in the affected breast could help unravel the mysteries underlying breast carcinogenesis, progression, recurrence, and resistance to treatment.

## Methods

### Sampling design

Surgically resected fresh-frozen tissues (*n* = 57) from therapeutic mastectomy specimens from primary breast cancer, risk reduction mastectomy (*n* = 5; three BRCA1/2 mutants, one BRCA1/2 wild type, one BRCA1/2 status unknown), and cosmetic reduction mammoplasty (*n* = 5) were obtained from the Barts Cancer Institute Breast Tissue Bank and Barts Health NHS Trust, London, UK. Ethics approval was obtained from the East of England - Cambridge Central Research Ethics Committee (approval no. 15-EE-0192), with written informed consent obtained from each patient.

Standard hematoxylin & eosin (H&E) stained slides were scored for relative proportions of benign, epithelial, stromal, and tumor cells to allow selection of appropriate sample cores from the patient cohort. Using fresh-frozen mounts, regions of tumor and matched tissues located proximal to (<2 cm) and distant from (5–10 cm) the tumor border were identified and marked for macro-dissection.

To confirm IHC staining for estrogen receptor (ER) and Her2, completed as standard of care by Barts and The London NHS Trust, validatory IHC staining with antibodies for ER (Abcam, ab16660, 1 μg/ml) and Her2 (Abcam, ab134182, 1 μg/ml) was performed on fresh tissue mounts using standard methods and acetone buffer. In situ hybridization for Her2 was performed only in two cases where IHC is equivocal. For completeness, IHC for progesterone receptor (Novocastra, NCL-PGR-312, 1 μg/ml) was also performed. The receptor status presented in the manuscript is the final classification.

Briefly, frozen sections were thawed at room temperature (RT) then fixed in ice-cold acetone for 5 min. Tissues were blocked with 1% bovine serum albumin (1X phosphate-buffered saline [PBS]) and incubated with primary antibody for 30 min at RT in blocking buffer. Appropriate secondary antibodies (1:200 1X PBS) were incubated for 40 min at RT. Tissues were rinsed and incubated with Vectastain ABC reagent (Vector Laboratories) for 30 min at RT, then rinsed and incubated with 3,3′-diaminobenzidine (DAB)/H_2_O_2_ until good contrast was achieved. Sections were rinsed and counter-stained with Mayers, rehydrated and mounted. These sections were scored (low/medium/high intensity) to determine each patient’s receptor status.

### Sequencing

Between 9 and 50 tissue sections (10 μm each, per patient and per site) were used for downstream RNA extraction of fresh-frozen tissues to obtain sufficient RNA for sequencing. Total RNA was extracted using the Qiagen RNeasy Plus Mini kit (Qiagen) according to the manufacturer’s protocol. RNA concentration was determined using the Qubit^®^ (Invitrogen) and RNA quality was assessed using Agilent Bioanalyzer 2.0 (Agilent Technologies); a minimum RIN threshold of 7.0 was applied.

RNA-seq libraries were prepared at the Wellcome Trust Centre for Human Genetics high throughput genomics unit using RNA-seq RiboZero Gold, according to the manufacturer’s instructions (Illumina). Paired-end reads of 150 bp in length were generated using the HiSeq 4000 (Illumina) platform, achieving just over 100 M paired-end reads per sample on average.

After checking FASTQ data quality with FastQC, raw reads were aligned to the reference genome hg38 using HISAT2 (https://ccb.jhu.edu/software/hisat2/index.shtml). The number of reads uniquely aligned (mapping quality score *q* > 10) to the exonic region of each gene were counted using the HTSeq package, based on the GENCODE annotation (version 23). Genes that achieved at least one count per million mapped reads in at least four samples were included (*n* = 19,472).

Read counts were normalized using the conditional quantile normalization method, which accounts for gene length and GC content. The log2-transformed reads per kilobase per million mapped reads (RPKM) were derived for all filtered genes across 67 samples. To account for batch effects, the *Combat* function via the SVA R package was applied (https://bioconductor.org/packages/release/bioc/html/sva.html). The corrected log2 expression values were adjusted to avoid negative values, as log2 (1 + unlogged expression value).

### Proteomics

Experiments were performed using mass spectrometry (MS) as described previously^47^. Briefly, fresh-frozen breast tissue cores from tumor, TP, and TD tissue (*n* = 33) were ground and lysed in urea lysis buffer (8 M urea, 10 mM Na_3_VO_4_, 100 mM β-glycerol phosphate, and 25 mM Na_2_H_2_P_2_O_7_) supplemented with phosphatase inhibitors (Sigma).

Proteins were digested into peptides using trypsin and phosphopeptides enriched from total peptides by TiO_2_ chromatography. Dried phosphopeptides were dissolved in 0.1% trifluoroacetic acid and analyzed by nanoflow ultimate 3000 RSL nano instrument, coupled to a Q-Exactive Plus mass spectrometer (ThermoFisher Scientific). Gradient elution was from 3 to 35% buffer B (0.1% formic acid in acetonitrile) in 120 min at a flow rate 300 nL/min, with buffer A (0.1% formic acid in water) used to balance the mobile phase. The spray voltage was 1.95 kV, with capillary temperature 255 °C. The Q-Exactive plus was operated in data-dependent mode with one survey MS scan followed by 15 MS/MS scans. The full scans were acquired in the mass analyzer at 375–1500 *m*/*z* with the resolution of 70,000, and the MS/MS scans were obtained with a resolution of 17,500.

MS raw files were converted into Mascot Generic Format using Mascot Distiller (version 2.5.1) and searched against the SwissProt database (December 2015 release), restricted to human entries using the Mascot search daemon (version 2.5.0). Allowed mass windows were 10 ppm and 25 mmu for parent and fragment mass-to-charge values, respectively. Variable modifications included in searches were oxidation of methionine, pyro-glu (N-term) and phosphorylation of serine, threonine, and tyrosine.

KSEA was implemented to infer kinase activity from phosphoproteomic data and provide formal determination of pathways enriched in the data^11^.

### Proteomics informed by transcriptomics

PIT analysis was performed using methods described previously^20^ to generate a list of translated genomic elements (TGEs) and classify them according to how they differ from the Uniprot canonical sequence. Default parameters: 1% peptide-spectrum match-level FDR; and peptide evidence ≥ *2*.

Profiles of translated genomic elements are presented as UpSet plots generated using UpSetR (v1.4.0).

### Data analysis

Genes were ranked based on median absolute deviance (MAD) of expression values, with the top 5000 most variable genes selected for PCA. PCA on tumor and normal profiles was conducted using the R package FactoMineR v1.41 (https://cran.r-project.org/web/packages/FactoMineR/index.html).

The intrinsic.cluster.predict function, available from the genefu package (https://www.bioconductor.org/packages/release/bioc/html/genefu.html), was called to fit a single-sample predictor to the data using the PAM50 classifier (sbt.model=pam50.scale)^8^. The probabilities of each subtype allocation were estimated using the Subtype Clustering Model and returned in the subtype.proba R value.

We selected the 5000 most variable genes (MAD) and applied the consensus NMF method to identify sample clusters^10^. Consensus matrices and sample correlation matrices were obtained for *k* = 2 to *k* = 8, with the optimal clustering solution determined by the most stable *k*-factor decomposition. NMF parameters: Brunet algorithm; *k* = 2–8 clusters; error function = Euclidean; number of clusterings to build consensus matrix=20; iterations = 500. Clustering patterns were confirmed using the R package ConsensusClusterPlus v1.38.0 (https://bioconductor.org/packages/release/bioc/html/ConsensusClusterPlus.html).

Differential expression analyses were conducted using EdgeR (https://bioconductor.org/packages/release/bioc/html/edgeR.html). A significance threshold of FDR ≤ 0.05 and a log-fold change of ≥1 was implemented during the differential analysis of each spatial group (tumor, TP, and TD) relative to RM.

To identify genes most representative of each cluster, we implemented the function *SAMseq* in the samr R library v2.0 (https://cran.r-project.org/web/packages/samr/index.html). SAMseq parameters: resp.type=“Multiclass”, nperms=100. The top gene sets representing each class were ranked further using the average expression values of each gene and de termining differences in the values between the represented cluster versus that of the non-represented cluster with the largest average value.

Enrichment analyses on genes differentially expressed between the spatial locations, relative to RM, and cluster-specific signatures were performed using the ClueGO Cytoscape Plugin^48^. ClueGO parameters for enrichment: Ontologies: GO (Biological Processes, Molecular Function, Cellular Component and Immune System Processes), Reactome^49^, KEGG; GO fusion; Evidence = Experimental and Inferred from the literature; Hierarchy depth = 5–10; significance threshold = *p*_adj_ ≤ 0.05 (Bonferroni); kappa score ≥ 0.6.

Gene set enrichment analysis (GSEA) was performed using the Broad Institute desktop application (http://software.broadinstitute.org/gsea/downloads.jsp).

The leukocyte signature matrix (Cibersort: LM22^21^) was used to infer immune phenotypes for the four subgroups. Single-sample gene set scores were obtained for each signature in the normalized gene expression matrix to determine the representation of immune cell types in each patient. The scores for each signature were summarized for each patient population group and mean centered to permit comparisons between groups. Parameters: signature matrix = LM22, mean = geometric mean.

### Validation

RNA-seq data for breast cancer primary tumors with matched normal samples (*n* = 108) was obtained from the TCGA.

Patients were stratified into risk groups based on their class allocation by Support Vector Machine (SVM) using the R package e1071 v1.6-8 (https://cran.r-project.org/web/packages/e1071/index.html). A tuning step was implemented to define optimal parameters. The SVM model was subsequently applied to the TCGA RNA-seq data for subtype allocation. SVM optimized parameters: sampling method = 10-fold cross validation; cost = 0.01.

A Cox proportional hazards regression model was applied to assess the prognostic capability of the subtypes, with *p*-values estimated using log-rank test. Survival modeling and Kaplan–Meier analyses were conducted using the survival package v2.41-3 (https://cran.r-project.org/web/packages/survival/index.html).

Univariate modeling was also applied to every gene defining the 414-gene classifier independently via median dichotomization of the mRNA abundance intensities. These genes were collated and ranked based on their prognostic potential.

### Reporting summary

Further information on experimental design is available in the Nature Research Reporting Summary linked to this paper.

## Supplementary information

Supplementary Information

Supplementary Data 1

Supplementary Data 2

Supplementary Data 3

Supplementary Data 4

Supplementary Data 5

Supplementary Data 6

Reporting Summary Checklist FLAT

## Data Availability

The transcriptomic data underlying this study are available from the European Genome-Phenome Archive (EGA) at https://identifiers.org/ega.study:EGAS00001004510^50^, with the transcriptomic findings available for top-level analyses from Breast Cancer Now Tissue Bank (BCNTB) bioinformatics (http://bioinformatics.breastcancertissuebank.org)^51^. BCNTB bioinformatics will allow researchers to conduct exploratory, investigative and interpretative analyses on the pre-processed data. Supplementary data and a metadata record complementing this paper are available on figshare at 10.6084/m9.figshare.12656702^52^.

## Code availability

The code is available from the corresponding author by request.
