# Supplementary material for: Characterization of four subtypes in morphologically normal tissue excised proximal and distal to breast cancer
Source: NPJ Breast Cancer. 2020 Aug 21;6:38. doi: 10.1038/s41523-020-00182-9 (PMC7442642; doi:10.1038/s41523-020-00182-9)
Supplement: Supplementary file 1 — Supplementary Information [file 41523_2020_182_MOESM1_ESM.pdf]

Supplementary Information

Characterization of four subtypes in morphologically normal tissue excised proximal and distal to breast cancer. Gadaleta *et al.* 2020

Supplementary Data Set 1 Clinical and pathological characteristics of the cohort.

Supplementary Data Set 2 PAM50 probability scores.

Supplementary Data Set 3 Gene classifier.

Supplementary Data Set 4 NCI pathways enriched and depleted in each subtype.

Supplementary Data Set 5 Non-coding RNAs defining the nc-enriched subtype.

Supplementary Data Set 6 Genes with prognostic potential in histologically normal tissues.

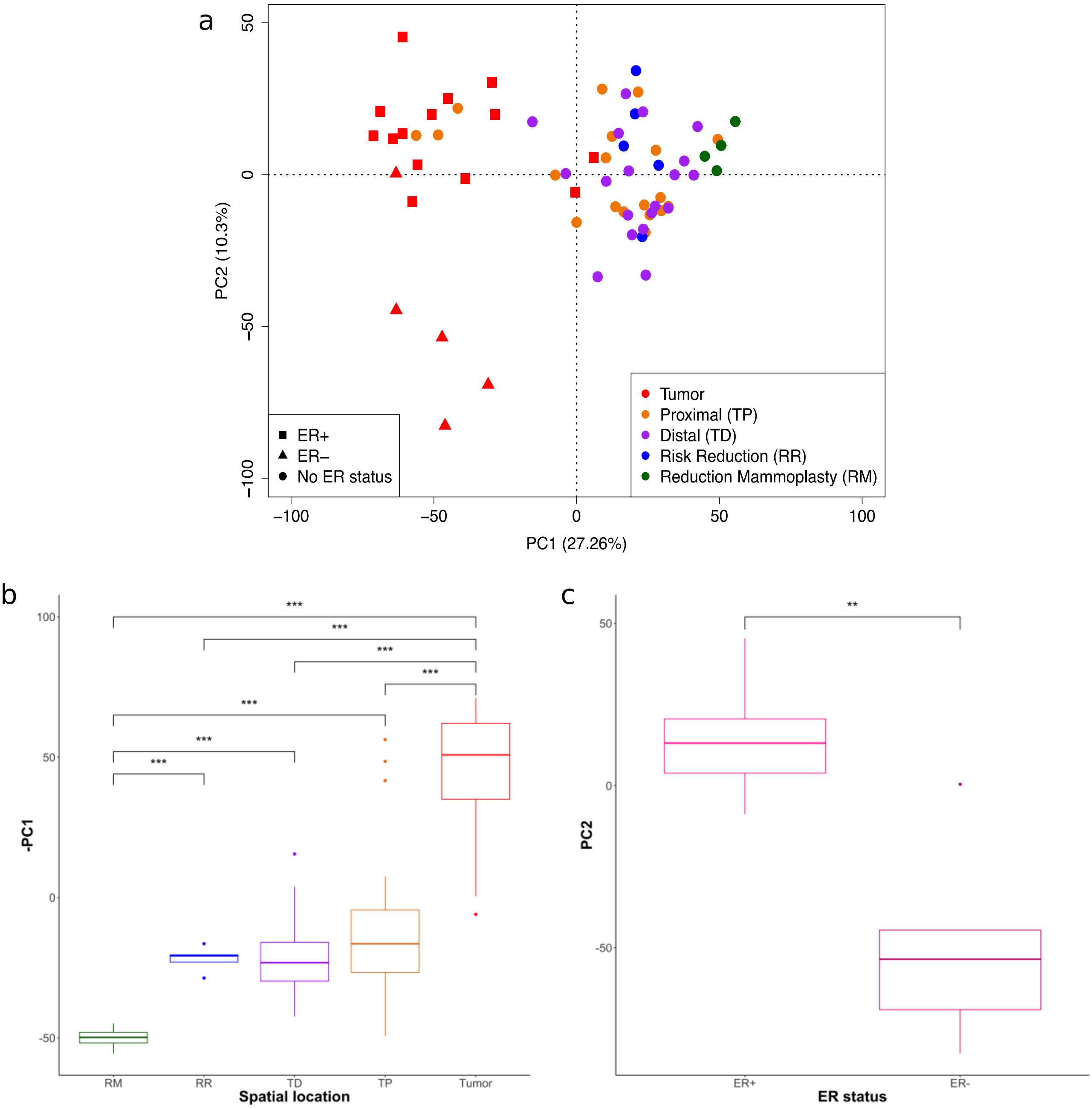
a
PC2 (10.3%)
PC1 (27.26%)
ER+
ER–
No ER status
Tumor
Proximal (TP)
Distal (TD)
Risk Reduction (RR)
Reduction Mammoplasty (RM)
b
-PC1
Spatial location
RM
RR
TD
TP
Tumor
c
PC2
ER status
ER+
ER-

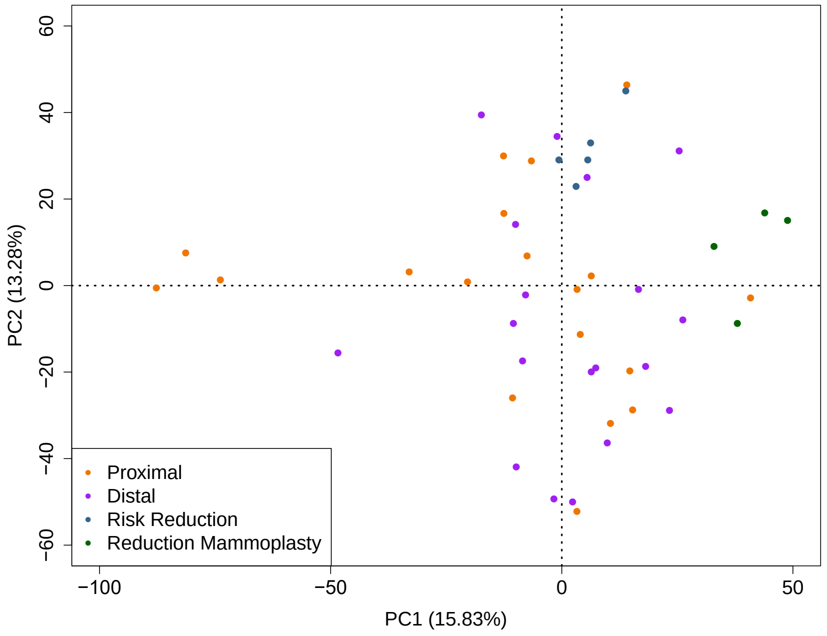

Supplementary Figure 1 PCA of the transcriptomic landscape of morphologically normal tissues. The transcriptomic landscape of breast cancer and morphologically normal tissues displays a wide dispersion of sample profiles across the main principal axes. **a,** Each point represents the orientation of a specimen projected into transcriptional space, with colors highlighting the relative location of the specimen. The shape of the tumor points represents ER status. Tumor profiles aggregate in distinct quadrants of transcriptional space, as do RR and RM. Profiles from HN tissues are interspersed and spread across all quadrants. **b,** There is a significant difference in the first PC between tumor and RM versus all other profiles. **c,** The spread of the data in the second PC is explained by ER status. **d,** There is a wide spread of proximal and distal profiles across the main principal axes. RR and RM specimens tend to exhibit homogeneity within their own groups, bar RM patient 2024.

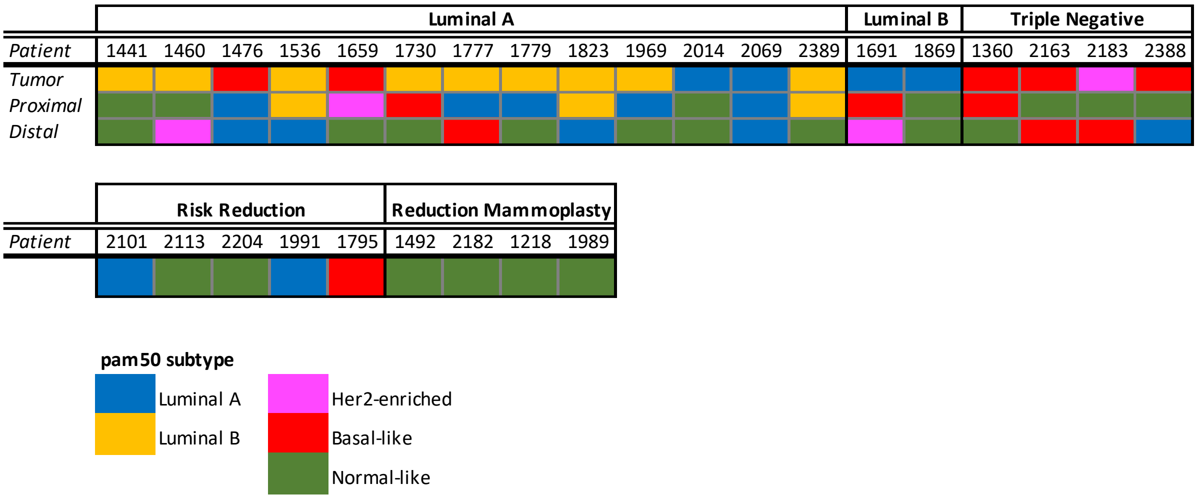

Supplementary Figure 2 Molecular classification of proximal and distal samples, with patients sorted based on the clinical classification of their primary tumor.

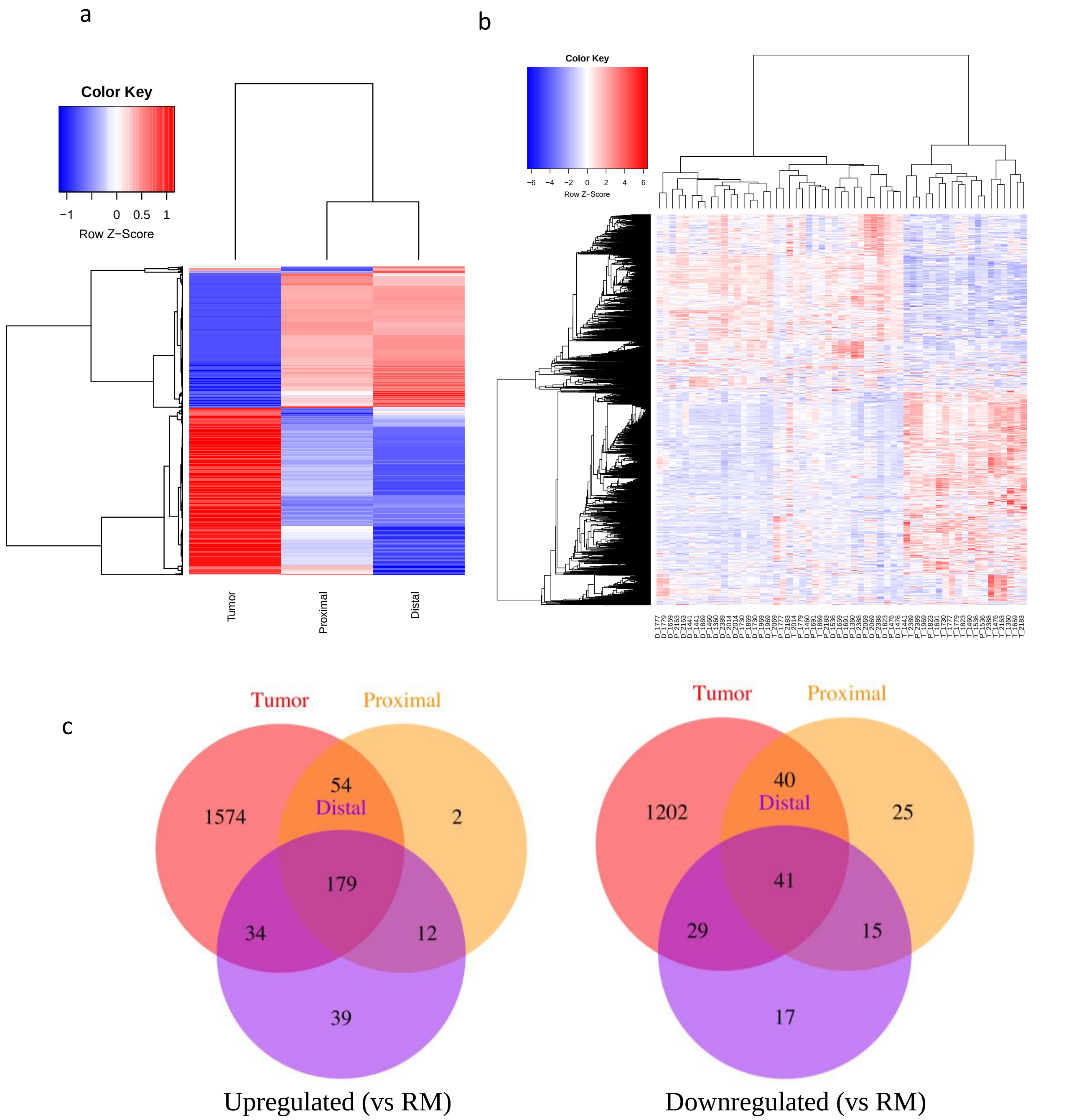

Supplementary Figure 3 Differential expression analysis of tumor, and matched normal tissues, using RM as baseline. **a,** Heatmap of the genes differentially expressed between each spatial group and RM shows dysregulation in tumor and matched tissues (FDR $\leq$ 0.05, log fold change $\geq$1). There is overlap in the differential expression profiles for the TP and TD groups. **b,** The individual-level heatmap reflects observations from the PCA. While tumors tend to cluster together, extratumoral specimens do not cluster based on their spatial location or patient. **c,** The total number of genes differentially expressed decreases as distance from primary tumor increases.

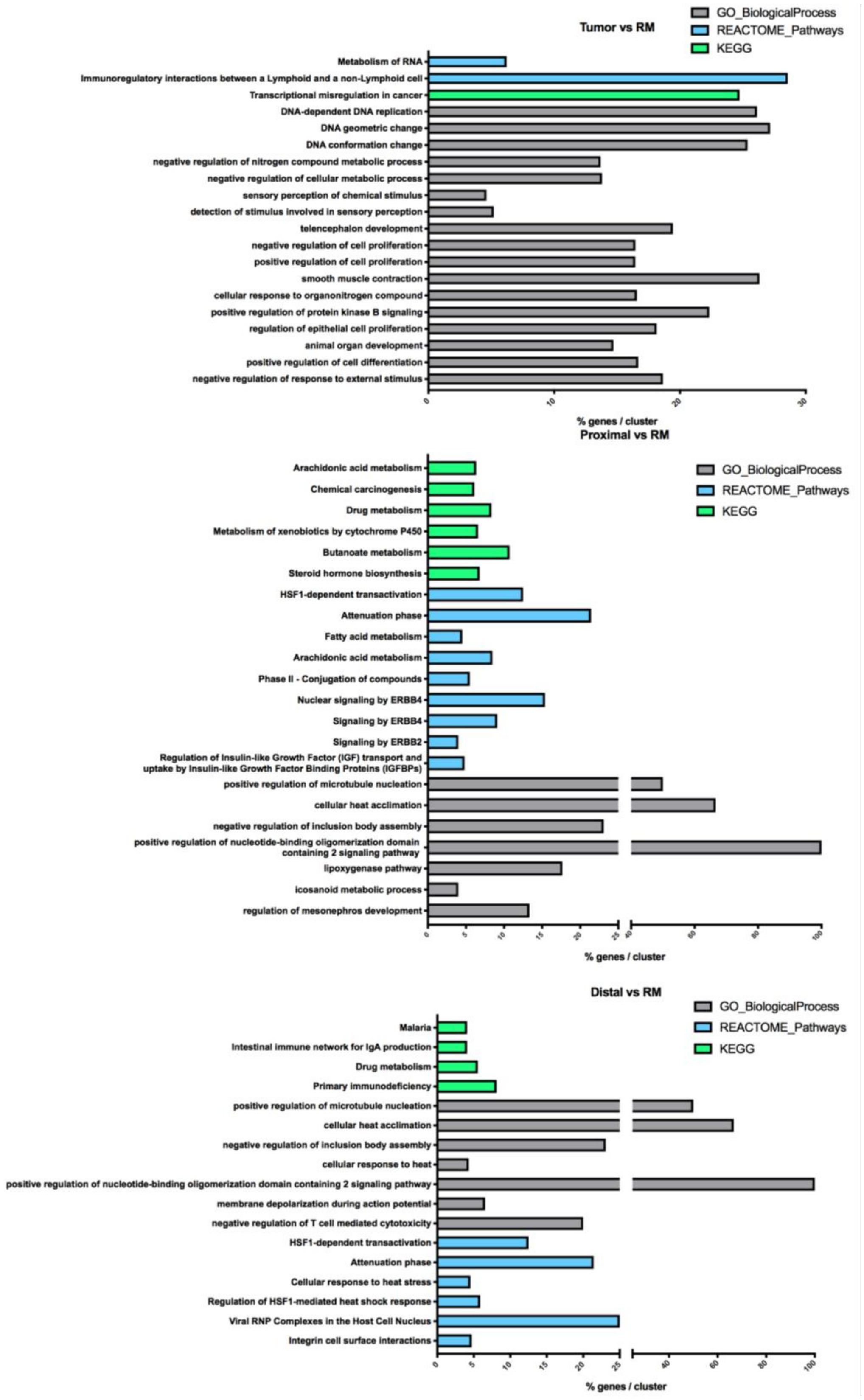

Supplementary Figure 4 GO terms and pathways enriched in each spatial group. The ClueGO Cytoscape plugin was employed to determine GO terms and pathways enriched in genes differentially expressed between each spatial group relative to RM ($p_{adj}<0.05$). More than 600 cancer-associated pathways were reported enriched in the tumor group. For clarity, the top 20 most significant results pertaining to this group are presented.

a

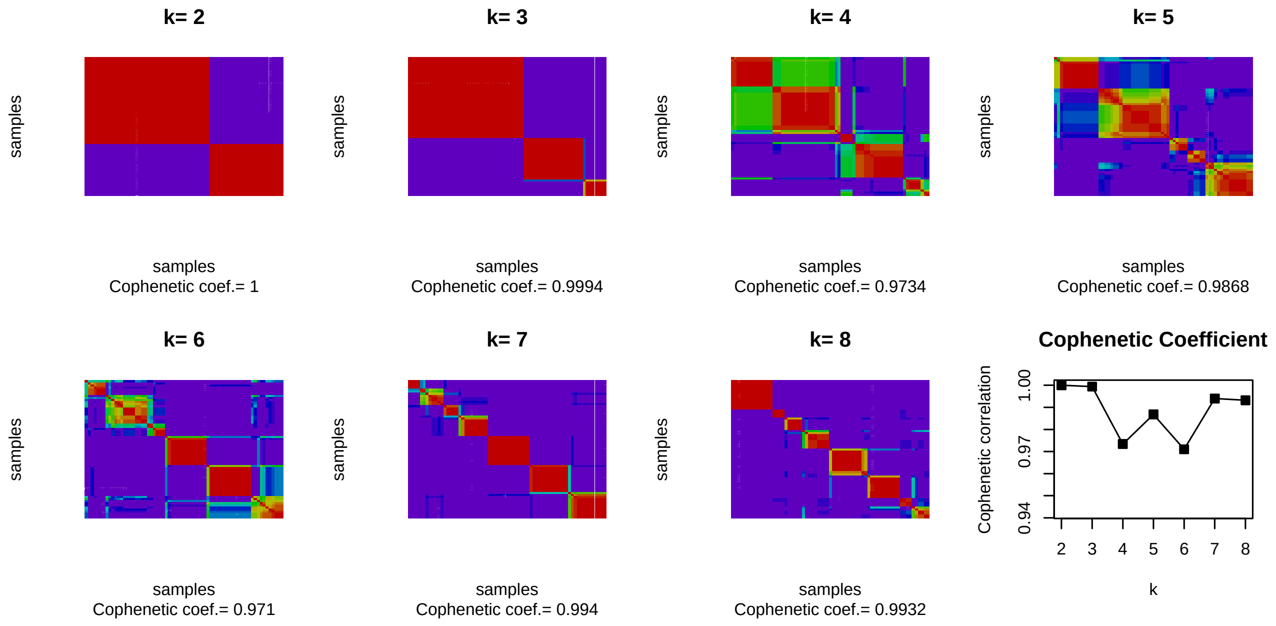

b

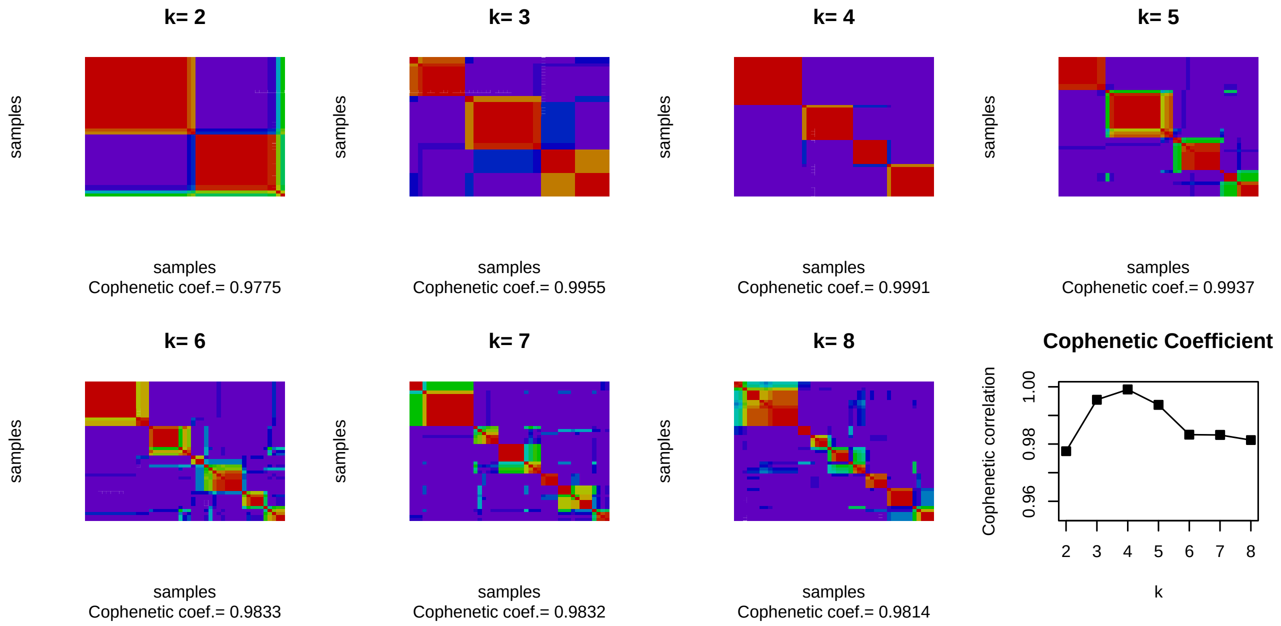

Supplementary Figure 5 NMF consensus clustering of the samples. **a,** Unsupervised NMF consensus clustering of tumor and HN samples for identify peak cophenetic coefficient for k=2 classes (cophenetic coefficient 1). **b,** NMF clustering of HN samples for solutions k=2 to k=8 identify four stable clusters (cophenetic coefficient 0.9991).

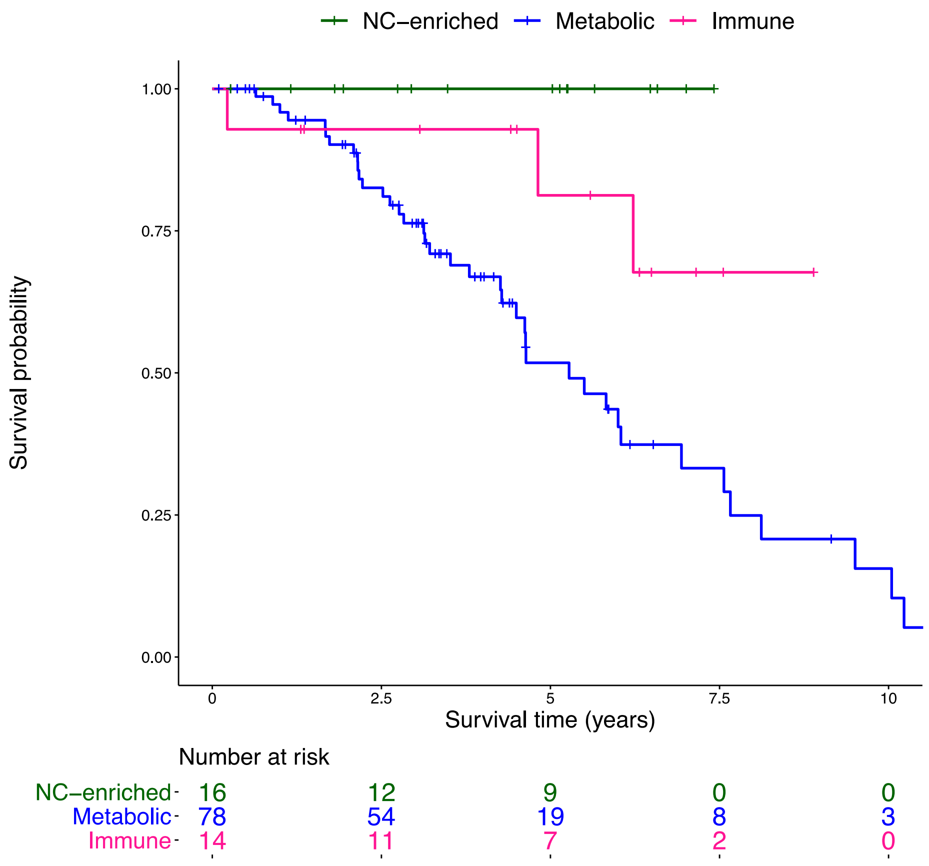

Supplementary Figure 6 Survival analysis indicates that the metabolic group has worse prognosis (log-rank p<0.001, hazard ratio 6.1) relative to the immune and the nc-enriched subtype. No events are present in the nc-enriched group. However, the small sample size prevents any conclusive interpretations to be made.
