# Supplementary material for: Characterization of four subtypes in morphologically normal tissue excised proximal and distal to breast cancer
Source: NPJ Breast Cancer. 2020 Aug 21;6:38. doi: 10.1038/s41523-020-00182-9 (PMC7442642; doi:10.1038/s41523-020-00182-9)
Supplement: Supplementary file 8 — Reporting Summary Checklist FLAT [file 41523_2020_182_MOESM8_ESM.pdf]

nature research

Corresponding author(s): Claude Chelala - 00579

Last updated by author(s): Apr 20, 2020

Reporting Summary

Nature Research wishes to improve the reproducibility of the work that we publish. This form provides structure for consistency and transparency in reporting. For further information on Nature Research policies, see Authors & Referees and the Editorial Policy Checklist.

Statistics

For all statistical analyses, confirm that the following items are present in the figure legend, table legend, main text, or Methods section.

n/a	Confirmed	
☐	☒	The exact sample size (*n*) for each experimental group/condition, given as a discrete number and unit of measurement
☐	☒	A statement on whether measurements were taken from distinct samples or whether the same sample was measured repeatedly
☐	☒	The statistical test(s) used AND whether they are one- or two-sided<br>*Only common tests should be described solely by name; describe more complex techniques in the Methods section.*
☐	☒	A description of all covariates tested
☐	☒	A description of any assumptions or corrections, such as tests of normality and adjustment for multiple comparisons
☐	☒	A full description of the statistical parameters including central tendency (e.g. means) or other basic estimates (e.g. regression coefficient) AND variation (e.g. standard deviation) or associated estimates of uncertainty (e.g. confidence intervals)
☐	☒	For null hypothesis testing, the test statistic (e.g. *F*, *t*, *r*) with confidence intervals, effect sizes, degrees of freedom and *P* value noted<br>*Give P values as exact values whenever suitable.*
☒	☐	For Bayesian analysis, information on the choice of priors and Markov chain Monte Carlo settings
☐	☒	For hierarchical and complex designs, identification of the appropriate level for tests and full reporting of outcomes
☒	☐	Estimates of effect sizes (e.g. Cohen's *d*, Pearson's *r*), indicating how they were calculated

Our web collection on statistics for biologists contains articles on many of the points above.

Software and code

Policy information about availability of computer code

Data collection	No software was used.
Data analysis	We used open source published software and code fully described in the manuscript with references provided.

For manuscripts utilizing custom algorithms or software that are central to the research but not yet described in published literature, software must be made available to editors/reviewers. We strongly encourage code deposition in a community repository (e.g. GitHub). See the Nature Research guidelines for submitting code & software for further information.

Data

Policy information about availability of data

All manuscripts must include a data availability statement. This statement should provide the following information, where applicable:

- Accession codes, unique identifiers, or web links for publicly available datasets
- A list of figures that have associated raw data
- A description of any restrictions on data availability

Raw data will be freely available from the EGA upon acceptance. Results will be available from the Breast Cancer Now Tissue Bank bioinformatics portal.

Field-specific reporting

Please select the one below that is the best fit for your research. If you are not sure, read the appropriate sections before making your selection.

☒ Life sciences ☐ Behavioural & social sciences ☐ Ecological, evolutionary & environmental sciences

For a reference copy of the document with all sections, see nature.com/documents/nr-reporting-summary-flat.pdf

Life sciences study design

All studies must disclose on these points even when the disclosure is negative.

Sample size	We performed a comprehensive computational analysis of breast cancers (n=19) and their matched HN tissues (n=38), healthy breast from cosmetic reduction mammoplasty (RM; n=5), and risk reducing mastectomies (RR, n=5), using a combination of RNA sequencing and proteomic profiling. Peritumoral samples were excised at proximal (TP, <2 cm) and distal (TD, 5-10 cm) sites from the primary tumor.
Data exclusions	NA
Replication	In the absence of publicly available datasets with similar sampling design and sufficient specimens, we selected 108 matched adjacent tissues from the TCGA BRCA dataset to validate our subtypes. Support vector machine (svm)-based modeling was applied to assign each TCGA adjacent sample to one of our transcriptomic subtypes. We were able to classify the TCGA samples into three of our four subtypes – metabolic, immune and nc-enriched. However, the matrisome/EMT subtype as defined by our study was not found within the TCGA cohort.
Randomization	NA
Blinding	NA

Reporting for specific materials, systems and methods

We require information from authors about some types of materials, experimental systems and methods used in many studies. Here, indicate whether each material, system or method listed is relevant to your study. If you are not sure if a list item applies to your research, read the appropriate section before selecting a response.

Materials & experimental systems

n/a	Involved in the study	
☒	☐	Antibodies
☒	☐	Eukaryotic cell lines
☒	☐	Palaeontology
☒	☐	Animals and other organisms
☐	☒	Human research participants
☒	☐	Clinical data

Methods

n/a	Involved in the study	
☒	☐	ChIP-seq
☒	☐	Flow cytometry
☒	☐	MRI-based neuroimaging

Human research participants

Policy information about studies involving human research participants

Population characteristics	See above
Recruitment	Breast Cancer Now Tissue Bank
Ethics oversight	Approval was obtained from the East of England - Cambridge Central Research Ethics Committee (approval no. 15-EE-0192), with written informed consent obtained from each patient.

Note that full information on the approval of the study protocol must also be provided in the manuscript.
